# Constant chemical potential approach for quantum chemical calculations in electrocatalysis

**DOI:** 10.3762/bjnano.5.79

**Published:** 2014-05-20

**Authors:** Wolfgang B Schneider, Alexander A Auer

**Affiliations:** 1 Max-Planck-Institute for Chemical Energy Conversion, Stiftstraße 34–36, D-45470 Mülheim an der Ruhr, Germany

**Keywords:** density functional theory, electrocatalysis, electrochemistry, electronic strutcture theory, nanoparticles, quantum chemistry

## Abstract

In order to simulate electrochemical reactions in the framework of quantum chemical methods, density functional theory, methods can be devised that explicitly include the electrochemical potential. In this work we discuss a Grand Canonical approach in the framework of density functional theory in which fractional numbers of electrons are used to represent an open system in contact with an electrode at a given electrochemical potential. The computational shortcomings and the additional effort in such calculations are discussed. An ansatz for a SCF procedure is presented, which can be applied routinely and only marginally increases the computational effort of standard constant electron number approaches. In combination with the common implicit solvent models this scheme can become a powerful tool, especially for the investigation of omnipresent non-faradaic effects in electrochemistry.

## Introduction

In October 2012 the workshop “Elementary reaction steps in electrocatalysis: Theory meets experiment” was held in Reisensburg, Germany. Alongside exquisite experimental work on electrochemistry, numerous prominent contributions displayed the range of modern developments and applications of theory in electrochemistry. This included the application of solid state approaches [1–4], investigations on the role of the solvent [5–9] or simulations including explicit dynamics of reactants [10]. Furthermore, several contributors presented work in which cluster models were applied in the framework of electronic structure theory in order to assess the properties of nanoparticles, nanostructures or interfaces [11–15].

Today, several approaches are available for modelling the full details of the electronic structure in electrochemical phenomena. The most common approach is to describe faradaic processes by using a thermodynamic scheme in which reaction energy differences are corrected a posteriori by the number of electrons transferred times the electrochemical potential the simulation is supposed to refer to [16–19]. This allows to monitor the changes in the system behavior depending on the electrochemical potential without having to include the electrochemical potential of the electrons in the calculation explicitly. As a consequence, this “pure thermodynamic” approximation, which is often also referred to as “computational hydrogen electrode” [16], and which we previously denoted as “constant charge approach” [13], allows to use the results of a single electronic structure calculation for all potentials [20–21]. Furthermore, this approach is also very convenient for periodic boundary calculations as in this case the models are restricted to a neutral unit cell.

However, there are several electrochemical phenomena for which it is clear that the explicit inclusion of the electrochemical potential is vital, namely the broad family of non-faradaic processes. This induces potential induced or lifted surface reconstructions [22] or the prominent non-faradaic electrochemical modification of catalytic activity (NEMCA) effect [23]. Only in recent years, attempts have been made to go beyond the pure thermodynamical approximation, explicitly including the electrochemical potential into the electronic structure calculation by means of adding or removing fractions of electrons or the introduction of electric fields included explicitly or via counter charges. For this reason, the effect of the explicit inclusion of the electrochemical potential in the electronic structure calculation for phenomena from electrocatalysis has yet to be quantified, and it is currently still open to debate if the pure thermodynamic approach is sufficient for certain processes.

Generally, electronic structure methods can roughly be divided in two subcategories, i.e., methods that treat the system within a unit cell by using periodic boundary conditions and methods that restrict the description of the system to the finite model chosen. In this work, we focus on finite systems approaches from quantum chemistry for treating electrochemical phenomena. These methods, especially in the framework of density functional theory (DFT), have in recent decades been applied for a broad variety of problems related to electrochemistry. This includes for example cluster models, that are applied to model reactions on surfaces [18–19,24], it includes the calculation of molecular properties to understand the redox properties of organic molecules and it includes the simulation of small to medium sized nanoparticles to explore their stability and the role of their atomic and electronic structure in electrocatalysis.

In a recent publication we have presented a constant potential scheme for calculating the electronic structure of a system at a given electrochemical potential [25]. This scheme is the quantum chemical equivalent to an approach by Alavi et al. [26], that focused on constant electrochemical potential schemes in the framework of periodic boundary condition DFT calculations. Based on the possibility to calculate the electronic structure of a finite system after adding or removing fractions of electrons, various quantities like the Fermi level (in that case the HOMO energy) or the numerical derivative of the energy with respect to the number of electrons can be used to associate a specific charge state of the system with its electrochemical potential. This, however, necessitates a complex computational scheme, for which several calculations have to be carried out in combination with an interpolation scheme that is far from the convenience of a black box application inherent to standard electronic structure calculations.

In the literature, only very few examples for constant potential schemes in the framework of quantum chemical approaches can be found: Bureau and Lécayon [27] describe the basic principles for devising an algorithm in which the target quantity is the chemical potential rather then the number of the electrons. After discussing the necessity and possibility to carry out constant-*μ* calculations, the authors lay out the theoretical underpinning in the framework of linear response theory and variational DFT schemes. They describe a variational procedure in which the Kohn–Sham equations are solved in the framework of a Grand-Potential approach with a variable number of electrons and a fixed *μ*. Finally, a scheme is proposed, in which a series of standard calculations with a given number of electrons are carried out and for each fixed electron number the chemical potential is evaluated afterwards. While this approach is conceptually simple, the computational effort can be immense if larger systems like nanoparticles are investigated [25]. Shiratori et al. [28–29] presented a scheme for carrying out constant-*μ* calculations based on a finite temperature Grand Canonical ansatz. They propose to optimize the wave function parameters explicitly including the chemical potential of the electrons, keeping the number of electrons variable through the SCF cycles. While this approach seems a promising solution for an algorithm to calculate the electronic structure of a system at a given potential, it has some pitfalls as we shall discuss in the following sections. Furthermore, Bonnet et al. showed that it is possible to calculate the properties of a system for a given potential in the framework of ab-initio molecular dynamics [30]. In this work, we present an algorithm that allows to calculate the electronic structure for a given system not with a fixed number of electrons, but with a given target chemical potential. We outline the problems of previously devised schemes and arrive at an algorithm that has the potential for a black-box scheme that can be applied for systems ranging from small molecules (insulators) up to metallic nanoparticles.

## Theory

In principle, there are several ways to evaluate the chemical potential *μ* for a given system: A rough estimate can be obtained by the negative of the electronegativity of Pauling and Mulliken [31], calculated by the ionisation potential (*I*) and electronegativity (*A*) of the system or its approximations by the orbital energies.

[1]
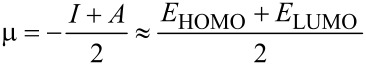


Furthermore, in calculating the free energy of a system, a Fermi–Dirac distribution function is applied to obtain the occupation numbers at a given temperature (“Fermi smearing”). Here, the chemical potential appears as a parameter for the Fermi smearing in the form of the Lagrangian multiplier for the number of electrons.

[2]
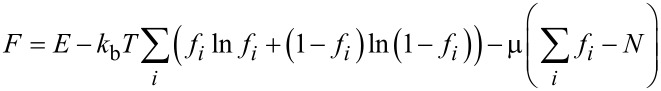


[3]
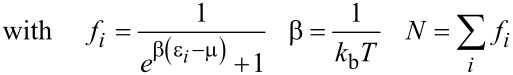


By definition, *μ* is the derivative of the energy with respect to the number of electrons (Equation 4). Hence, it can be evaluated either analytically (for example as an analytic derivative or by linear response theory) [27] or numerically by calculating *E* for various electron numbers [26].

[4]
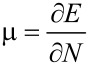


At this point the basic difference between a Canonical and a Grand Canonical Ensemble should be emphasized. In a Canonical Ensemble, a constant number of electrons is assumed for each micro system, while the chemical potential is an average over the micro systems. In a Grand Canonical Ensemble the chemical potential is constant for each micro system and the number of electrons per micro system is an average. In this context, constant charge calculations as typically carried out in electronic structure theory can be attributed to a Canonical Ensemble ansatz at zero temperature. This can be extended to finite temperatures (Equation 2) by using a Fermi–Dirac distribution for the electronic degrees of freedom. This introduces fractional occupation numbers and the chemical potential of the electrons as a Lagrangian multiplier that ensures a constant number of electrons in the treatment. The Grand Canonical approach differs from this by fixing the chemical potential of the system while allowing electron exchange with an external bath.

[5]
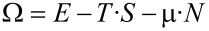


Bureau et al. [27] showed, that the chemical potential and the corresponding number of electrons obtained by calculating the free energy of a Canonical Ensemble equals the values that are obtained by calculating the grand potential (Equation 5) of the corresponding Grand Canonical Ensemble. Thus, the free energy and the Grand Potential can easily be converted (Equation 6).

[6]
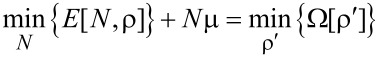


By calculating the electronic structure of an oxygen atom for different fractional numbers of electrons, Vuilleumier et al. showed that the three approaches yield comparable results for the calculation of the electrochemical potential [32]. Hence most of the constant potential schemes are derived from calculations with a constant number of electrons. An iterative procedure to directly calculate the energy of a system depending on the chemical potential was for example discussed by Shiratori et al. [28]: After converging the energy with an initial number of electrons, the number of electrons is changed by Δ*N* and a new value for *μ* is obtained. This procedure is carried out until a converged wave function is obtained at the desired value of the chemical potential. The disadvantage of this approach is that the number of iterations needed can be fairly high. Thus, the approach is associated with a considerable computational overhead.

In principle, an algorithm for the iterative calculation with varying number of electrons and given chemical potential can be constructed based on Equation 2. Instead of using the chemical potential as a parameter to guarantee a constant number of electrons, it is possible to directly insert the aspired potential and obtain the number of electrons for that given potential. This is a very convenient way of determining a Δ*N*: After the new occupation numbers *f_i_* have been obtained for the new number of electrons, the density matrix is modified by using Equation 3.

[7]
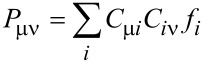


**Figure 1:**
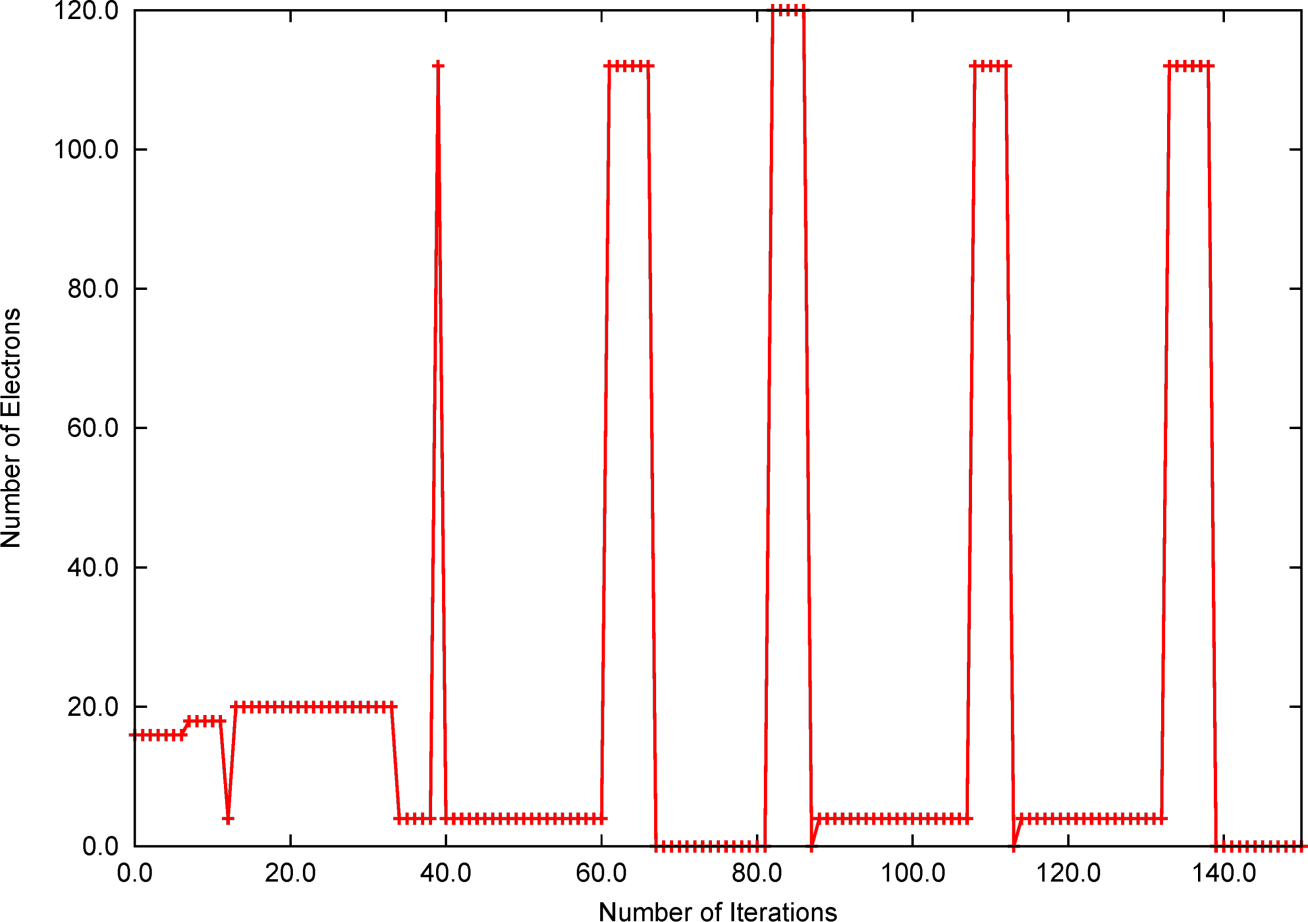
Evolution of the number of electrons with the number of iterations for O_2_ if the potential dependent energy is computed by inserting the aspired *μ* into Equation 2.

Next, the energy is again converged followed by another modification of the density matrix until convergence of the number of electrons (and thus the chemical potential) and the energy is achieved. However, while this scheme is appealing, the crucial point is the convergence of the overall scheme. A robust algorithm is essential in any case, for simple systems like molecules and especially for complex examples like metallic nanoparticles. In Figure 1 the number of electrons with the number of SCF iterations is monitored if the scheme discussed above is applied to calculate the electronic structure of the O_2_ molecule at an absolute potential of −3.71 V. Note that the absolute potential of the charge neutral O_2_ with a bond distance of 1.21 Å calculated at the RI-BP86/def2-TZVP level of theory is −5.71 V. For all calculations in this paper the following convergence parameters were applied: The energy was converged up to 10^−9^ a.u., the maximal density change up to 10^−5^, RMS density change up to 10^−6^ and the DIIS error up to 10^−6^ a.u. Furthermore, all calculations have been carried out without level shift for the virtual orbitals.

After the convergence of the initial charge state with 16 electrons is achieved, the number electrons is slightly increased. However, in the further course of the calculation the number of electrons oscillates between the minimum of 0 electrons and the maximum of 120 electrons. Hence, no convergence is observed, even for this very simple case.

**Figure 2:**
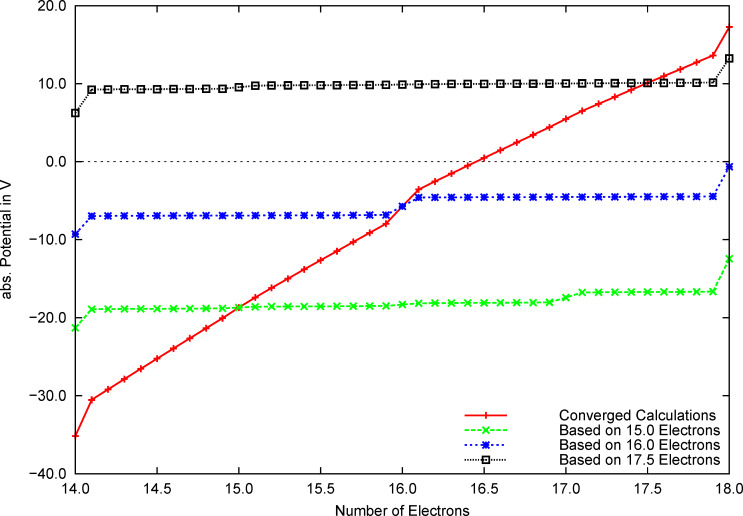
Change of the absolute potential for O_2_ depending on the number of electrons, calculated numerically and approximated by Equation 3, respectively.

The origin of this behavior is revealed in Figure 2, in which the dependence of the potential on the number of electrons is plotted according to Equation 2. For the red/solid exact curve, the potential was obtained by Equation 2 from converged calculations using fractional number of electrons and hence, is the exact *μ*(*N*). The orbital energies *ε_i_* obtained from calculations with a *N* of 15, 16 and 17.5 electrons were used to approximate *μ*(*N*) by Equation 3. The slope calculated numerically by fractional charge states for O_2_ shows a strong dependence of the potential on the number of electrons. However, the slope of *dμ*/*dN* calculated by the approximation using Equation 3 is much smaller (dashed green, blue and black line). This leads to a drastic overestimation of the change of the charge and causes erratic steps in the optimization of the charge of the system, impeding convergence of the algorithm.

A better approximation to obtain *dμ*/*dN* is the calculation of a new density matrix based on the old MO coefficients 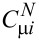 but with a changed number of electrons by using Equation 8. This new density matrix is used to calculate a new Fock matrix and an approximated new energy (Equation 9).

[8]
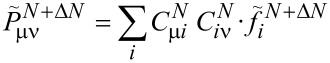


[9]
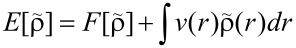


**Figure 3:**
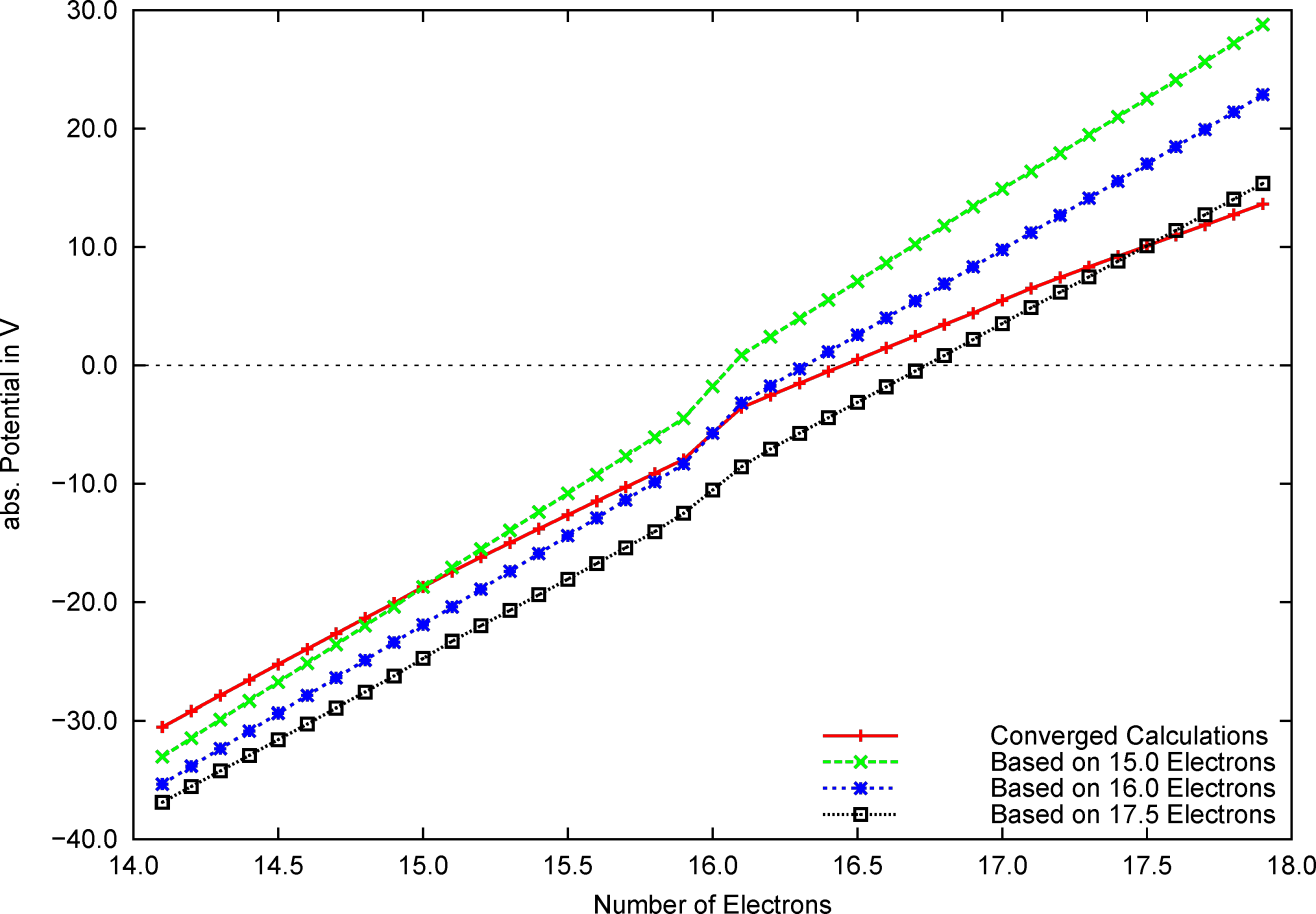
Chemical potential of the O_2_ molecule, plotted against the number of electrons, calculated numerically and approximated by recalculation of the Fock matrix, respectively.

In Figure 3 the dependence of the potential on the number of electrons is plotted for O_2_ by using this new approximation. Similar to Figure 2, the red/solid curve corresponds to the numerically calculated *μ*(*N*) (Equation 4) of converged calculations using a fractional *N*. Using the MO coefficients of the converged calculations with a *N* of 15.0, 16.0 and 17.5 electrons, approximated energies were calculated by using Equation 9. The approximated values of *μ* were obtained by numerical differentiation and are plotted with dashed lines. As can be seen, the approximation based on the recalculated Fock matrices yields a much better approximation for the exact slope than the approximation based on the Fermi smearing formula. Moreover, the approximated slope is always larger than the exact *dμ*/*dN*, circumventing the overestimation of the change with the number of electrons that was observed in the previous approximation.

**Figure 4:**
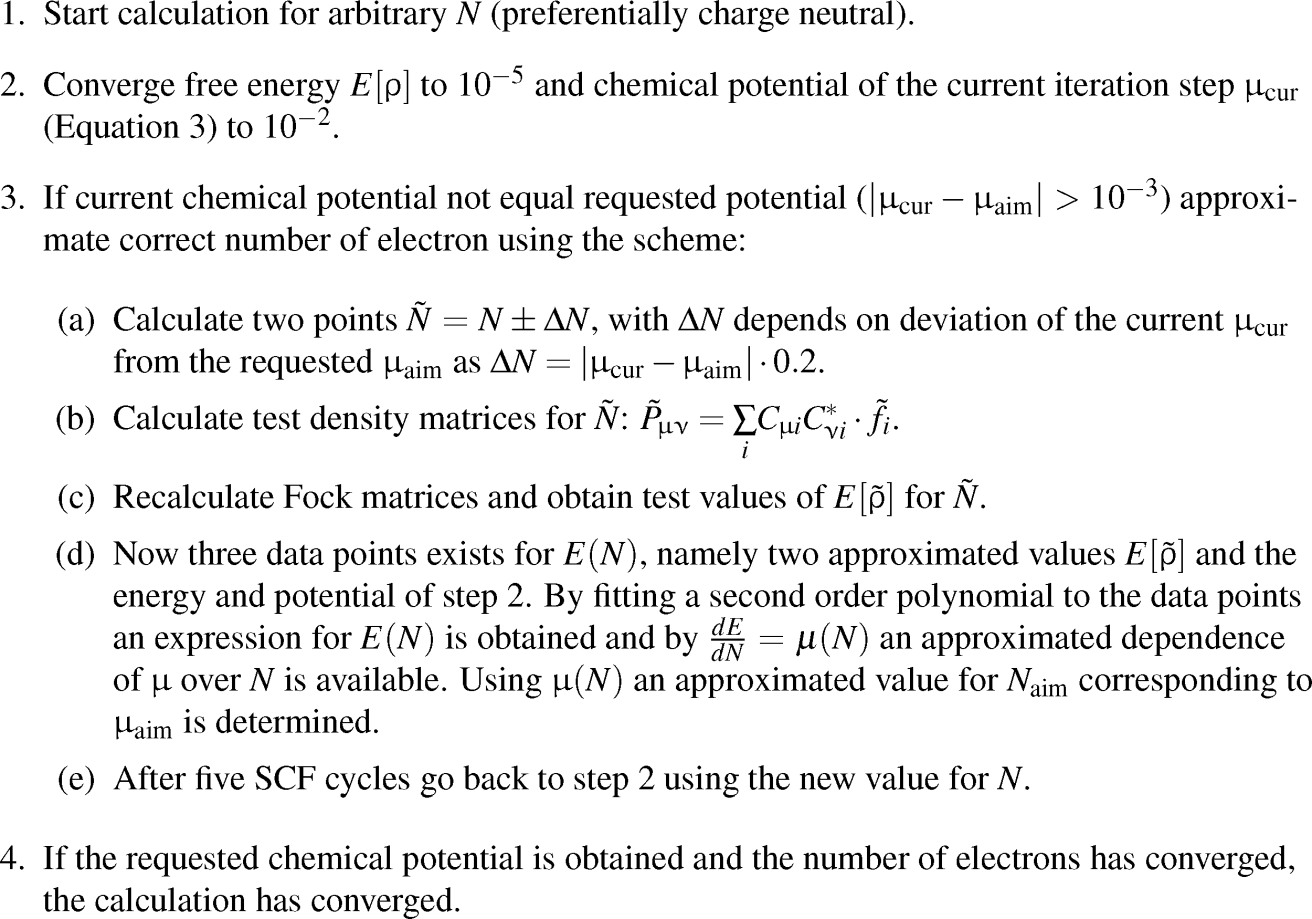
Scheme for a potential dependent calculation of the free energy.

As the function *E*(*N*) exhibits a quadratic dependence on the energy, its derivative can easily be evaluated by a three-point scheme. For this purpose, the Fock matrix and the corresponding energy is calculated for three different points using the MO coefficients obtained for the current number of electrons. Assuming a quadratic form, an approximation for *∂*^2^*E*/*∂N*^2^ is obtained, which is then used to predict the number of electrons for the target chemical potential. Note that as a consequence, the computational costs of an iteration step approximately triple. However, it is not necessary to calculate a new number of electrons in every iteration, as several tests on smaller and larger model systems show that it is sufficient to converge the SCF equations to a certain extend by using a fixed number of electrons and only to adjust it every few iterations depending on the degree of convergence. Based on this, the scheme shown in Figure 4 was applied to a testset of molecules.

The potential obtained in this way is the absolute potential with the electron at rest in the vacuum as reference. It can be related to the experimentally achieved potential by a constant shift using the Trasatti scheme [33]. It should be noted that in actual applications, for which constant potential calculations will yield different numbers of electrons for the same system in different states, reaction enthalpies need to be calculated by adding the corresponding *e*·*U* correction, as discussed in a previous work [25].

Some final remarks about the validity of the overall scheme should be made at this point. The scheme presented here is a Grand Canonical Ensemble DFT approach that relies on a proper response of the system with respect to change in the number of electrons. It can be argued that typical functionals might not be well suited for this purpose. In molecular systems, for example, ionization potentials and electron affinities are often not well reproduced. DFT yields a continuous function of *μ* over *N*, though a step function is expected. Furthermore, the use of fractional electrons in the description of the system, as inherent to this approach, is not consistent with the ideas of quantized charge transfer in a real molecular system of isolated active sites on a surface. However, metallic systems at finite temperature with high or infinite density of states and small or vanishing bandgap, show a continuous change of the potential with the number of electrons. Furthermore, in the framework of a Grand Canonical approach the treated system is in contact with a bath of electrons, which models the situation of a subsystem in contact with a conducting environment. Thus, while limited in applicability, the approach is well suited for the treatment of metallic nanoparticles on conductive supports or within cluster approaches to model surface reactions. The developed algorithm has been implemented in the ORCA [34] programme package.

## Calculations on test systems

**Figure 5:**
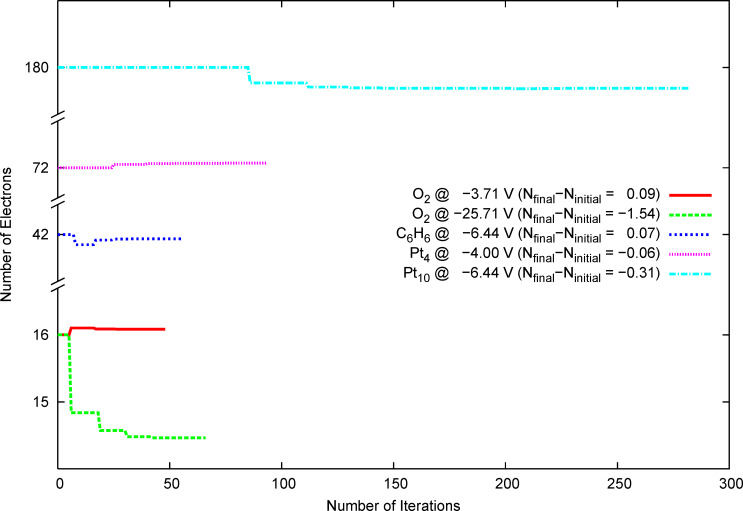
Convergence of the number of electrons with the SCF iterations for different systems. Note that the calculation for the charge neutral molecules with fixed number of electrons for O_2_ converges within 7, for C_6_H_6_ within 13, for Pt_4_ within 73 and for Pt_10_ within 177 SCF iterations, respectively.

Using the new scheme, the above mentioned calculation for oxygen at a potential of −3.71 V was repeated. As shown in Figure 5, already the first approximation of the final number of electrons is fairly good (solid red curve). This is the case, even if a huge deviation of the requested chemical potential from the initial chemical potential exists. For O_2_ at an extreme absolute potential of −25.71 V good convergence on 14.4 electrons is achieved within six updates of the number of electrons (dashed green curve). This is just one update more, compared to the calculation at −3.71 V.

The new scheme was tested for further examples like small organic molecules and metallic clusters (Figure 5). For all examples fast convergence of the number of electrons for the given potentials was achieved. For benzene, calculated at the RI-BP86/aug-ccPVTZ level of theory, convergence of the absolute potential and the energy was observed after 55 iterations. For the metal clusters Pt_4_ and Pt_10_, calculated at the RI-BP86/def2-TZVP level of theory, convergence was observed after 92 (−4 V) and 238 SCF iterations, respectively. Standard calculations for small molecules like oxygen or benzene using fixed number of electrons converge within 10–20 SCF iterations. If the calculations are carried out at fixed potential, the number of SCF iterations is approximately quintupled. However, systems with increasing metallic character, such as platinum clusters, show a slower convergence for a fixed number of electrons (50–70 iterations). For these systems, the number of SCF iterations approximately doubles if the calculation is carried out at fixed potential. This can be compared to a previous work [25] in which the energy of platinum clusters at a given potential was determined by an interpolation scheme. There, it is necessary to calculate the energy of the system at least for three different numbers of electrons in order to obtain a result for a given potential. In total, this amounts to (at least) threefold computational effort and hence the computational effort is reduced by using the new scheme.

One important aspect to note is the correlation between the calculated energy and the convergence of the absolute potential. Depending on the convergence criterium for the potential, the number of electrons *N* is also only converged with a certain error. For example, if the energy of O_2_ is calculated at *μ* = −4 V, starting with 16 or 15 electrons, respectively, the final number of electrons differs by 3.6·10^−5^, if the potential is converged to 10^−3^ V. However, as the energy strongly depends on the number of electrons, this leads to a deviation of 0.01 kJ/mol in the final energies.

For systems such as metallic structures, for which *μ*(*N*) has a smaller slope than for oxygen, the resulting error in *N* for a given potential is larger, and hence the error of the calculated energy increases. For instance, if the energy of a Pt_4_ cluster is calculated at 1000 K over a potential range of −16 V to 4 V, with an initial charge of 0 and −1, respectively, the final energies for the same potential can differ by up to 5.0 kJ/mol, if the potential is converged up to 10^−3^ V. While for most purposes this error can be controlled by choosing the appropriate convergence criteria and consistent starting points for the calculation, the user should be aware of this behavior.

## Conclusion

In this work, an SCF iteration scheme to calculate the electronic energy of a system at constant electrochemical potential in the framework of a Grand Canonical Ensemble DFT ansatz is presented. In contrast to common DFT calculations, that are carried out at a constant number of electrons *N*, the energy is calculated for a fixed electrochemical potential with a variable fractional number of electrons.

While earlier approaches require the calculation of the energy for different *N* [25–27], in the scheme presented here, the optimization of *N* is incorporated in the SCF iterations of the energy calculation. For this purpose it is decisive to find a good approximation for *dμ*/*dN* in order to obtain a good guess for the final *N*. The simple approach of estimating the correct *N* by inserting the requested *μ* into the Fermi–Dirac distribution function used in finite temperature DFT leads to an oscillatory behavior of *N* during the SCF iterations. A much better and still computationally simple approximation of *N*_final_ is obtained by numerical evaluation of *dμ*/*dN*, recalculating the Fock matrix for different values for *N* during an additional update step. This way, a robust and efficient algorithm is obtained to carry out constant potential calculations in the framework of quantum chemical approaches.

Whether this model can be routinely applied in the computation of faradaic and non-faradaic electrochemical processes has to be subject to careful benchmarks, which is work in progress and will be published in forthcoming articles.
